# Tomographic Evaluation of the Lower Incisor's Bone Limits in Mandibular Symphysis of Orthodontically Untreated Adults

**DOI:** 10.1155/2017/9103749

**Published:** 2017-10-17

**Authors:** Paula Guerino, Mariana Marquezan, Maurício Barbieri Mezomo, Kaline Thumé Antunes, Renésio Armindo Grehs, Vilmar Antônio Ferrazzo

**Affiliations:** ^1^Department Stomatology, Universidade Federal de Santa Maria, Santa Maria, RS, Brazil; ^2^Department of Orthodontics, Centro Universitário Franciscano, Santa Maria, RS, Brazil

## Abstract

The amount of available bone in the lower incisor region is critical for periodontal preservation when planning large anteroposterior dental movements. The aims of this study were to evaluate bone limits of the lower incisors in the mandibular symphysis and to verify whether they are influenced by facial growth patterns, lower incisor inclinations, skeletal anteroposterior relationships, or patient age. Tomographic images of 40 orthodontically untreated patients were evaluated and measurements of width and height of the mandibular symphysis, thickness on the lingual and labial sides of the alveolar bone, and thickness of the entire alveolar bone were performed in sagittal view. The following cephalometric measurements were also evaluated: growth pattern (FHI), lower incisor inclination (IMPA), and skeletal anteroposterior relationships (AO-BO). Pearson's correlation test was used to assess associations among bone measurements, cephalometric measurements, and patients' ages. Weak to moderate positive correlations between FHI and bone measurements on the labial side of the incisors and total alveolar width were found. The height of the symphysis had a moderate negative correlation with FHI. It was concluded that patient age, FHI, and IMPA influenced bone limits of the lower incisors in the mandibular symphysis, while AO-BO had no influence.

## 1. Introduction

The morphology of the mandibular symphysis and the position of the lower incisors are crucial factors for the success of orthodontic treatment [1–6]. Investigation of mandibular bone structures in the lower incisor region can aid in determining initial tooth position [3], direction of the orthodontic tooth movement, and occlusal stability at the end of orthodontic treatment [7].

The amount of available bone in the lower incisor region must be considered when planning large anteroposterior dental movements [8], such as in cases with premolar extractions [9, 10], distalization using temporary anchorage devices [11], or execution of compensatory orthodontic treatment with large compensation [3, 12]. Care must be taken to avoid problems that affect periodontal support and protection, such as dehiscence, bone fenestration, and gingival recession [3, 5, 9, 10].

Cone-beam computed tomography (CBCT) images are highly reliable [13] and, hence, make it possible to analyze the thickness and level of the bone plates covering the teeth on the labial and lingual sides [14–16]. Although the gold standard method for evaluation of the bone plates is tomographic imaging, the current recommendation of the AAOMR (American Academy of Oral and Maxillofacial Radiology) is that CBCT be indicated for cases where its use will be critical to the establishment of the diagnosis and/or treatment plan, weighing the risks and benefits of the additional radiation [17]. When conventional radiographs requiring less radiation can be used to obtain the necessary data, CT scans should be avoided. However, lateral radiographs, traditionally used in orthodontic documentation, are less reliable for evaluating the buccolingual thickness of the alveolar process in the lower incisor region due to image overlapping [2, 5, 14, 18, 19]. Therefore, efforts have been made to establish associations between certain dentofacial characteristics and bone thickness in the mandibular symphysis region in an attempt to predict the quantity of bone tissue in this region [2, 5, 7, 12, 20, 21].

The aims of this study were (1) to determine the thickness and height of the mandibular symphysis in the lower incisor region in orthodontically untreated adults; (2) to determine the thickness of the labial and lingual bone walls around the lower incisor roots; and (3) to evaluate the associations between these measurements and facial growth patterns, lower incisor inclinations, skeletal anteroposterior relationships, and patient age.

## 2. Materials and Methods

The CBCT scans of 40 patients treated at a private orthodontic clinic (Proprium Dentistry, Santa Maria, Brazil) were evaluated. The research protocol was approved by the Research Ethics Committee (CEP) of the Federal University of Santa Maria (Santa Maria, RS, Brazil; CAAE: 53310316.0.0000.5346).

All tomographic image exams were obtained with a Gendex GX CB-500 tomograph (Gendex Dental Systems, Hatfield, PA, USA) with standard settings (120 kVp, 5 mA, acquisition time of approximately 23 seconds, and a field of view that was 14 cm in diameter × 8 cm in height with 0.25 mm voxels). Both male and female adult patients were considered for inclusion. The inclusion criterion was to have four erupted lower incisors. The exclusion criteria included the following: patients who had previously undergone orthodontic or prosthetic treatment, present syndromes, or history of periodontal disease and poor image quality (artifacts or distortions).

For tomographic evaluation, the images in Digital Imaging and Communications in Medicine (DICOM) format were imported into OsiriX (OsiriX Foundation, Geneva, Switzerland). Initially, a multiplanar reconstruction (MPR) was performed to obtain an image corresponding to a cephalometric radiograph profile, and facial growth pattern was determined by analyzing the facial height index (FHI) [22], which is the ratio of the posterior facial height (PFH) to the anterior facial height (AFH) [23]. On the basis of FHI, facial growth patterns were classified as follows: hyperdivergent, FHI values lower than 0.649; normal, FHI values between 0.65 and 0.75; and hypodivergent, FHI values greater than 0.751. The same image was used to determine the inclination of the most projected lower central incisor by using the angle of inclination of the most projected lower incisor (IMPA) [1]. The skeletal anteroposterior relationship was determined by Wits analysis (AO-BO) [24].

**Figure 1:**
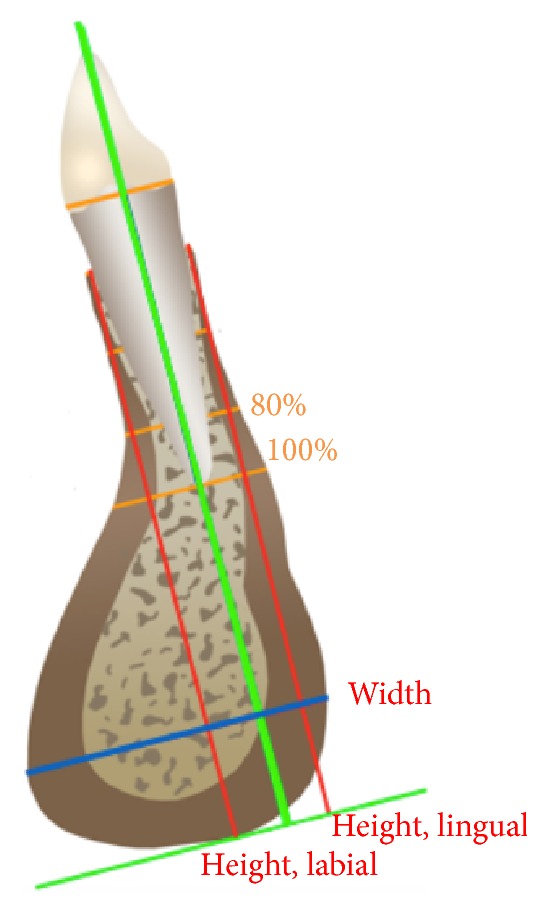


To perform bone thickness measurements, MPRs were carried out through the center of the lower incisor root canal to obtain sagittal views that corresponded to the central portions of the lower incisors. A root canal image was used as a reference to standardize tracing of the long axis of the lower incisor. Root length was defined and measured as the distance from the cementoenamel junction to the apex. A line perpendicular to the long axis of the incisor was used to establish reference points: 0% of the root represented the cement enamel junction and 100% represented the apex. The following measurements were made in the sagittal section of the tomographic image: labial alveolar bone thickness, lingual alveolar bone thickness, total alveolar bone thickness, total mandibular symphysis thickness, and total mandibular symphysis height on the labial and lingual sides of the lower incisors (Figure 1).

Measurements of alveolar bone thickness on the labial and lingual sides of the lower incisor roots were performed in two predetermined locations. Lines perpendicular to the long axis of the lower incisor were drawn at 80% and 100% of root length. To determine the height of the entire mandibular symphysis, a line parallel to the long axis of the tooth was drawn from the point representing the bony base of the lower incisor to a line perpendicular to the long axis of the tooth and traced at the lowest point of the cortical bone of the mandibular symphysis on the labial and lingual sides. For mandibular symphysis thickness determination, a line was drawn perpendicular to the long axis of the tooth in the thickest portion of the mandibular symphysis (Figure 1).

The spatial resolution of the scans was determined by using an acrylic phantom (Gendex Dental Systems, Hatfield, PA, USA). The phantom's tomographic image was acquired with the same specifications as the patients' scans and in accordance with the manufacturer's recommendations. The spatial resolution of tomographic images was 0.7 mm.

Repeating measurements twice for 20% of the sample with a 1-week interval between each evaluation assessed intraexaminer agreement and the results were excellent (ICC > 0.9) [25].

Statistical Package for the Social Sciences 20 (SPSS Inc., Chicago, IL, USA) was used for statistical analysis. Normality was analyzed by the Shapiro-Wilk test and correlations between bone measurements, FHI, IMPA, AO-BO, and patient age were analyzed using Pearson's correlation test.

Out of 40 patients, 70% were female; 62.5% were skeletal Class I, 15% were Class II, and 22.5% were Class III. The mean age was 34.2 (±14.6) years.

## 3. Results

The alveolar bone thickness measurements at different positions along the root lengths of the lower incisors are expressed in Table 1.

Associations between bone thickness in the region of each incisor, patient age, and the cephalometric measurements (FHI, IMPA, and AO-BO) are presented in Tables 2–5. Weak to moderate positive correlations were observed between FHI and bone measurements on the labial side of the incisors and total measurement of alveolar width. However, on the lingual side, no associations were found. The height of the symphysis had a weakly positive correlation with patient age and a moderately negative correlation with FHI. The total symphysis width showed a positive correlation with IMPA. IMPA also showed an association with age: there was a weakly negative correlation between IMPA and age. Age also influenced the width of alveolar bone (80%) in the region of tooth 42 (weakly negative correlation). Variations in the sagittal skeletal relationship (AO-BO) were not correlated with bone thickness in the lower incisor region.

## 4. Discussion

This study verified that the bone thickness of the lower incisor region increased from 80% of root length to the apical portion of the root, as was found by Nauert and Berg [4]. This can be attributed to the anatomy of the mandibular symphysis, in drop form.

As the FHI increased, the labial bone thickness at 80 and 100% of root length also increased, but bone height decreased in the labial and lingual regions. Thus, one assumes that a dolichofacial or hyperdivergent patient presents with a thinner and longer alveolar process in the lower incisor region but that a patient with a brachyfacial growth pattern has a thicker and shorter alveolar process. This result is in agreement with that reported by Handelman [3] and Swasty et al. [21], who showed that patients with longer faces tended to present with thinner bone structure at all of the measured mandibular sites. Gracco et al. [2] also observed greater labial bone thickness at the root's apex level in brachyfacial patients. Tsunori et al. [12] found an association between the FHI and labial cortical bone in the mandibular symphysis. Handelman [3] also found greater labial bone thickness in the lower incisors of patients with short faces.

This study also verified that labial bone thickness is greater than lingual bone thickness in the apex region, a finding that indicates that the root apex of the lower incisor is closer to the lingual side, in accordance with the observations of Farret et al. [26]. This fact must be considered when planning labial or lingual inclination movements in the lower incisors.

No association was found between facial growth patterns and lingual alveolar bone thickness, which is in agreement with the findings of Tsunori et al. [12] and Swasty et al. [21].

Considering the height of the symphysis, this study confirmed the findings of other studies that show that a dolichofacial pattern is associated with greater bone height (longer symphysis) [5, 12, 21].

The total bone thickness of the mandibular symphysis, measured at the thickest portion of the mandibular symphysis and perpendicular to the tooth's long axis, was not significantly associated with facial growth patterns, skeletal anteroposterior relationships, or patient age. These results corroborate those reported by Tsunori et al. [12].

In this study, there was no association between the skeletal anteroposterior relationships and bone measurements in the lower incisor region. Several studies [3, 27–29] show that Class III skeletal patients have thinner bones; however, according to Chung et al. [28], other factors associated with a skeletal Class III relationship, such as the vertical relationship between the anterior teeth, may influence bone thickness in the lower incisors.

Patient age influenced symphysis height and IMPA. With increasing age, an increase in symphysis height and a decrease in IMPA were observed. These findings can be attributed to continuous growth of alveolar bone and late growth of the mandible, which promotes incisor retroinclination. In contrast, Garcia et al. [27], after analyzing a sample of lateral cephalometric radiographs, found no relationship between the thickness of the alveolar process in the anterior mandible and patient age.

A positive association was observed between the IMPA and the total thickness of the mandibular symphysis. The thicker symphysis may have allowed greater movement of the lower incisors in patients who had not undergone orthodontic treatment.

Our findings demonstrate that a careful analysis of each individual's bone condition must be performed prior to determining a treatment plan, especially when considering a large extent of movement. It is believed that patients with a more vertical growth pattern (with lower FHI) require more stringent standards for buccolingual movement of the lower incisors than do patients with other facial patterns.

It is important to note that image quality limits the ability to perform linear measurements on CBCT images. The quality of a CBCT image, represented by its spatial resolution, depends on factors such as scanner settings, patient position, and voxel size [19, 30]. Ballrick et al. [31] claim that poor spatial resolution can make it impossible to detect differences between two small objects. According to Sun et al. [32], reducing the voxel size from 0.4 mm to 0.25 mm would be suitable for analyzing small structures with better precision and would improve the accuracy of linear measurements on CBCT scans. In this study, the acrylic scanned phantom demonstrated a spatial resolution of 0.7 mm. Measurements smaller than 0.7 mm should be observed with caution. For this reason, measurements obtained in more cervical portions of the incisor root were not considered.

There are several limitations in this study. Due to the FOV size used (14 × 8 cm), cephalometric measurements that use the anterior cranial base as a reference, such as FMA and S-N.Go-Gn, could not be performed. Therefore, the evaluations of growth patterns and anteroposterior maxillary relationships were performed through FHI and AO-BO. Dental crowding was not evaluated. In addition, a larger sample size would provide stronger evidence. Further studies using larger FOV and sample size are required.

## 5. Conclusions

Significant individual variation was observed in the measurements of bone limits of the lower incisors and mandibular symphysis. It was observed that facial growth patterns, lower incisor inclinations, and patient age influenced bone limits of the lower incisor in the mandibular symphysis; however, skeletal anteroposterior relationships showed no influence.

## Figures and Tables

**Table 1:** Mean and standard deviation (SD) of alveolar bone thickness measurements at different positions along the root lengths of lower incisors.

	Tooth 42	Tooth 41	Tooth 31	Tooth 32
Measures, labial (mm)				
80%	1.95 (0.97)	1.97 (1.34)	2.02 (1.26)	1.82 (0.94)
100%	5.07 (1.96)	4.72 (2.42)	4.7 (2.05)	4.69 (1.65)

Measures, lingual (mm)				
80%	2.27 (1.05)	1.19 (0.83)	1.7 (0.81)	2.11 (1.06)
100%	3.68 (1.19)	3.63 (1.22)	3.31 (1.11)	3.68 (1.13)

Measures, total (mm)				
80%	7.62 (1.58)	7.07 (1.42)	6.89 (1.42)	7.4 (1.46)
100%	8.76 (2.09)	8.36 (2.17)	8.09 (2.01)	8.37 (1.97)
Width	14.85 (2.34)	15.19 (2.09)	14.99 (2.19)	14.64 (2.51)
Height, labial	28.74 (3.19)	28.70 (3.32)	28.85 (3.2)	28.94 (3.15)
Height, lingual	28.55 (3.16)	28.62 (3.36)	28.73 (3.1)	28.9 (2.97)

**Table 2:** Pearson's correlation for bone thickness measurements of tooth 42.

	FHI	AO-BO	Age
Measures, labial (mm)			
80%			
*r*	**.382** ^*∗*^	.164	−.198
*p*	.015	.313	.221
100%			
*r*	**.382** ^*∗*^	.165	−.234
*p*	.015	.308	.146

Measures, lingual (mm)			
80%			
*r*	.216	−.236	−.233
*p*	.181	.142	.148
100%			
*r*	.078	−.302	−.094
*p*	.633	.058	.565

Measures, total (mm)			
80%			
*r*	**.402** ^*∗*^	−.022	−**.337**^*∗*^
*p*	.010	.893	.033
100%			
*r*	**.403** ^*∗*^	−.017	−.273
*p*	.010	.915	.088
Width			
*r*	.175	.089	−.119
*p*	.281	.584	.464
Height, labial			
*r*	−**.397**^*∗*^	.104	**.318** ^*∗*^
*p*	.011	.524	.046
Height, lingual			
*r*	−**.405**^*∗*^	.109	**.327** ^*∗*^
*p*	.009	.503	.040

^*∗*^Statistically significant difference (*p* ≤ 0.05).

**Table 3:** Pearson's correlation for bone thickness measurements of tooth 41.

	FHI	IMPA	AO-BO	Age
Measures, labial (mm)				
80%				
*r*	.366^*∗*^	.243	.130	−.217
*p*	.020	.131	.424	.180
100%				
*r*	.404^*∗*^	.258	.135	−.207
*p*	.010	.108	.406	.199

Measures, lingual (mm)				
80%				
*r*	.001	−.056	−.189	.070
*p*	.996	.731	.244	.668
100%				
*r*	.016	−.027	−.168	.053
*p*	.921	.869	.301	.744

Measures, total (mm)				
80%				
*r*	.375^*∗*^	.182	.041	−.188
*p*	.017	.260	.801	.244
100%				
*r*	.460^*∗*^	.284	.056	−.201
*p*	.003	.076	.732	.213
Width				
*r*	.282	**.450** ^*∗*^	.117	−.269
*p*	.078	.004	.474	.093
Height, labial				
*r*	−.448^*∗*^	.034	.162	.268
*p*	.004	.834	.317	.095
Height, lingual				
*r*	−.425^*∗*^	−.024	.140	.344^*∗*^
*p*	.006	.885	.390	.030

^*∗*^Statistically significant difference (*p* ≤ 0.05).

**Table 4:** Pearson's correlation for bone thickness measurements of tooth 31.

	FHI	IMPA	AO-BO	Age
Measures, labial (mm)				
80%				
*r*	**.373** ^*∗*^	.157	.203	−.155
*p*	.018	.334	.210	.339
100%				
*r*	**.405** ^*∗*^	.300	.220	−.162
*p*	.010	.060	.173	.318

Measures, lingual (mm)				
80%				
*r*	−.006	−.003	−.229	−.133
*p*	.971	.983	.154	.412
100%				
*r*	.024	.023	−.268	−.134
*p*	.881	.888	.095	.411

Measures, total (mm)				
80%				
*r*	**.353** ^*∗*^	.274	.065	−.256
*p*	.025	.087	.689	.111
100%				
*r*	**.428** ^*∗*^	.308	.076	−.240
*p*	.006	.053	.639	.136
Width				
*r*	.199	.312	.204	−.201
*p*	.218	.050	.206	.213
Height, labial				
*r*	−**.451**^*∗*^	−.130	.163	.294
*p*	.003	.423	.314	.065
Height, lingual				
*r*	−**.416**^*∗*^	−.158	.123	**.336** ^*∗*^
*p*	.008	.331	.448	.034

^*∗*^Statistically significant difference (*p* ≤ 0.05).

**Table 5:** Pearson's correlation for bone thickness measurements of tooth 32.

	FHI	AO-BO	Age
Measures, labial (mm)			
80%			
*r*	.368^*∗*^	.159	−.157
*p*	.020	.327	.333
100%			
*r*	.388^*∗*^	.141	−.204
*p*	.013	.385	.207

Measures, lingual (mm)			
80%			
*r*	.157	−.129	−.225
*p*	.333	.428	.163
100%			
*r*	.087	−.248	−.207
*p*	.593	.122	.200

Measures, total (mm)			
80%			
*r*	.334^*∗*^	.002	−.300
*p*	.035	.989	.060
100%			
*r*	.376^*∗*^	−.024	−.290
*p*	.017	.883	.070
Width			
*r*	.047	.138	−.213
*p*	.772	.396	.187
Height, labial			
*r*	−.342^*∗*^	.088	.323^*∗*^
*p*	.031	.591	.042
Height, lingual			
*r*	−.349^*∗*^	.114	.342^*∗*^
*p*	.027	.484	.031

^*∗*^Statistically significant difference (*p* ≤ 0.05).
